# The Expression of Osteopontin and Wnt5a in Articular Cartilage of Patients with Knee Osteoarthritis and Its Correlation with Disease Severity

**DOI:** 10.1155/2016/9561058

**Published:** 2016-07-31

**Authors:** Yusheng Li, Wenfeng Xiao, Minghua Sun, Zhenhan Deng, Chao Zeng, Hui Li, Tuo Yang, Liangjun Li, Wei Luo, Guanghua Lei

**Affiliations:** ^1^Department of Orthopaedics, Xiangya Hospital, Central South University, Changsha, Hunan 410008, China; ^2^Department of Orthopaedics, Affiliated Hospital, Logistics University of Chinese People's Armed Police Forces, Tianjin 300162, China; ^3^Department of Joint Surgery, Changsha Central Hospital, Changsha, Hunan 410004, China

## Abstract

*Objectives*. This study is undertaken to investigate the relation between osteopontin (OPN) and Wnt5a expression in the progression and pathogenesis of osteoarthritis (OA).* Methods*. 50 cartilage tissues from knee OA patients and normal controls were divided into four groups of severe, moderate, minor, and normal lesions based on the modified grading system of Mankin. Immunohistochemistry and real-time PCR were utilized to analyze the OPN and Wnt5a expression in articular cartilage. Besides, the relations between OPN and Wnt5a expression and the severity of OA were explored.* Results*. OPN and Wnt5a could be identified in four groups' tissues. Amongst the groups, the intercomparisons of OPN expression levels showed statistical differences (*P* < 0.01). Besides, the intercomparisons of Wnt5a expression degrees showed statistical differences (*P* < 0.05), except that between the minor and normal groups (*P* > 0.05). The scores of Mankin were demonstrated to relate to OPN expression (*r* = −0.847, *P* < 0.01) and Wnt5a expression in every group (*r* = −0.843, *P* < 0.01). Also, a positive correlation can be observed between the OPN and Wnt5a expression (*r* = 0.769, *P* < 0.01).* Conclusion*. In articular cartilage, the expressions of OPN and Wnt5a are positively related to progressive damage of knee OA joint. The correlation between Wnt5a and OPN might be important to the progression and pathogenesis of knee OA.

## 1. Introduction 

As a highly universal and disabling illness, osteoarthritis (OA) has a commensurate tremendous individual and societal burden [1]. OA pathogenesis is caused by an imbalance between anabolic and catabolic factors, resulting in homeostasis disturbance of articular cartilage, which plays a vital role in OA [2]. Osteopontin (OPN), referred to as bone sialoprotein 1 (BNSP or BSP-1), is a multifunctional phosphoprotein secreted by a number of cell kinds, for instance, lymphocytes, macrophages, osteoclasts, vascular smooth muscle cells, and epithelial cells [3, 4]. OPN has been proved to be an important intrinsic regulator which is significant to the progression of OA [5]. Major researches have demonstrated that the expression level of OPN in synovium fluid and OA cartilage are in accordance with severity of inflammatory status and joint lesion of disease [6–8]. However, controversy still exists concerning the exact functional role of OPN in cartilage degradation [5].

Wnt proteins affect cellular homeostasis by regulating cell proliferation, cell fate determination, and differentiation [9]. Wnt5a is a representative of the Wnt family that activates the *β*-catenin-independent pathway. A number of research lines have shown that the articular joints development, including cartilage, bone, and joints cavities, is highly dependent on Wnt signaling. Historically, Wnt signaling pathways were divided in the pathway of *β*-catenin-dependent canonical Wnt signaling as well as various *β*-catenin-independent noncanonical pathways [10–12]. Most of the data concerning the role that Wnt signaling plays in OA is related to the canonical pathway [13, 14]. Additionally, many researches with conditional activation or inhibition of Wnt signaling in the cartilage show effects on disease activity [15–17].

Previous observations have revealed that both OPN and Wnt5a are inflammation factors secreted by macrophage. OPN is associated with *β*1-integrin and matrix metalloproteinase (MMP) in human chondrocytes [18]. *β*1-integrin can upregulate Wnt5a and induce the expression of MMPs [19]. We hypothesized that, in articular cartilage, OPN and Wnt5a might be related to the severity of disease in patients with knee OA. So as to study the assumption, the expression levels of OPN and Wnt5a in various levels of damaged samples of cartilage from human-beings with OA could be determined and the correlation between OPN and Wnt5a can be analyzed. The current research aimed to offer a more overall comprehension of OPN and Wnt5a in OA.

## 2. Materials and Approaches

### 2.1. Subjects

This study contained 10 normal healthy individuals and 40 patients at the age of 52–81 with major knee OA. If the radiological and clinical statistics met the requirements of the American College of Rheumatology and were thoroughly checked to exclude any type of secondary OA or other inflammatory joint illnesses, including rheumatoid arthritis and any other kinds of arthritis [20], OA patients were regarded eligible. This research has been verified by the Ethics Committee of Xiangya Hospital, Central South University, and all the patients offered informed consent (grant number: 201212063). The samples of osteoarthritic cartilage were gathered from the 40 patients with main OA with knee arthroplasty. The normal samples could be gained from the knees of 10 age matched postmortem donors, without any history of joint pain. Informed consent of ethics could be gained from all the families and donors.

### 2.2. Histology

Biopsies (bone/cartilage samples) could be gained from the medial and lateral sides of tibia plateau, including the zone of loading. With a cartilage surface of around 2.0 × 0.5 cm, the bone/cartilage samples (1.0 cm thick) were incubated in a freshly prepared paraformaldehyde, later dehydrated in a grading xylene and ethanol concentration and eventually embedded in paraffin. Representative paraffin-embedded and formalin-fixed tissue blocks were sectioned and retrieved for histological and immunohistochemical research.

After eosin and hematoxylin (HE) and safranin-O staining, a light microscopy was used to evaluate the histological changes of the sections. Then severity of OA was quantified with the usage of the modified grading score of Mankin [21]: severe lesions: ≥9; moderate lesions: 5–8; minor lesions: 1–4; and normal: 0. 12 samples (≥9), 17 samples (5–8), 18 samples (1–4), and 13 samples (0) were included in this study. Every specimen was evaluated by a “blinded” observer for two times and the approach of the two scores was applied in all the analytical studies.

### 2.3. Immunohistochemistry

The sections were deparaffinized, treated with 3% hydrogen peroxide for 10 min, and microwaved in 10 mmol/L citrate buffer (pH 6.0) to uncover the epitopes. Afterwards, the sections were incubated with the antibody of OPN at a 1 : 150 dilution for 1 h. After cleansing, a Fab polymer conjugate/horseradish peroxidase (PicTure*™*-Plus kit; Zymed Life Technologies, Carlsbad, CA, USA) was used to the sections for half hour. Lastly, the sections were incubated for 5 min with diaminobenzidine for signal development. A negative control was prepared simultaneously by neglecting the major antibody. The sections were evaluated by a pathologist who did not know anything about clinical statistics. The sections were checked under an Olympus microscope (magnification: ×100; Olympus Corporation, Tokyo, Japan) in order to access the OPN expression. Positive OPN immunostaining could be defined as immunoreactivity that can be detected in the perinuclear or other cytoplasmic areas of the chondrocytes. In the cartilage tissue, the relative distribution of OPN was quantified and visualized as mean grey values. Semiquantitative evaluation of the average grey values for the expression of OPN could be performed on the scanned autoradiograms utilizing Image J software and medical image analysis software- (MIAS-) 4400. A region from the surface of cartilage to the junction of cartilage-bone was analyzed and the grey-scale images could be captured and transferred to the absorbance units. The experiment had been repeated for three times and densities could be normalized against those with PBS. In order to decrease the error incurring from the small change in the thickness of section, three sections for every sample in total were averaged and assessed. Thus, the ultimate statistics which have been used in all the analytical studies were constituted of the average value of three independent assessments which stood for the mean degrees of OPN in the articular cartilage. The coefficient of the OPN expression changes was <2% in the articular cartilage.

For immunostaining of Wnt5a, a DAKOCSA kit (DAKO, Carpinteria, CA) was used according to the manufacturer's recommendations. The aforementioned process was carried out in the same way to evaluate the expression of Win5a.

### 2.4. Real-Time PCR

Total RNA was extracted from tissues of the bone/cartilage samples using the RNAprep Pure Tissue Kit (TIANGEN, Beijing, China) according to the manufacturer's protocol. After extraction, total RNA was converted into cDNA by reverse transcription reaction, and real-time-PCR was performed using the ABI 7500 Fast Real-Time PCR system (Applied Biosystems, Foster City, CA). The mRNA expression levels were evaluated and normalized to *β*-actin as an endogenous reference. Primers used were as follows: OPN: F-TCGTCTCAGGCCGTTGCA and R-CATCTGTTGTGGAGGGGTAGGT; Wnt5a: F-TGTGGTTTAATGGTGCCTGA and R-TTCGTCGTGCTCAAGGTATG; *β*-actin: F-GGAAATCGTGCGTGACATTA and R-GGAGCAATGATCTTGATCTTC.

### 2.5. Statistical Analyses

Static grey analysis from the MIAS was used to evaluate the average grey values in 10 random selected areas of the Wnt5a and OPN immunohistochemical slices. SPSS software for Windows (version 19.0; IBM SPSS, Armonk, NY, USA) was utilized for data analysis and management. One-way analysis of variance (ANOVA) was applied to study the difference between multiple groups in the mean values between multiple groups. Then Spearman's correlation and linear regression were conducted to determine the correlations between the mean grey values for Wnt5a and OPN in the articular cartilage and the OA Mankin score. The results are shown as the mean ± standard error of the mean. *P* < 0.05 was regarded to show a statistically considerable difference.

## 3. Results

### 3.1. Histology and Immunohistochemistry

**Figure 1:**
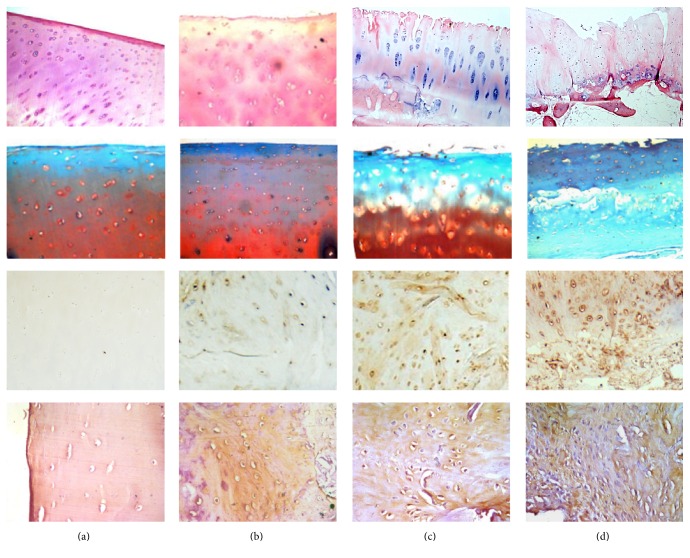
Histology and immunohistochemistry analysis. The first and second lines of images presented histological characteristics of the OA cartilage by HE staining and safranin-O staining, respectively. The third and fourth lines of images presented OPN and Wnt5a immunohistochemical staining, respectively. (a) In normal cartilage; (b) in minor lesions cartilage; (c) in moderate lesions cartilage; (d) in severe lesions cartilage.

The microscopic images of osteoarthritic changes in HE staining, safranin-O staining, OPN immunohistochemical staining, and Wnt5a immunohistochemical staining were presented in Figure 1.

### 3.2. Validation of OPN and Wnt5a Expression by Real-Time PCR

**Figure 2:**
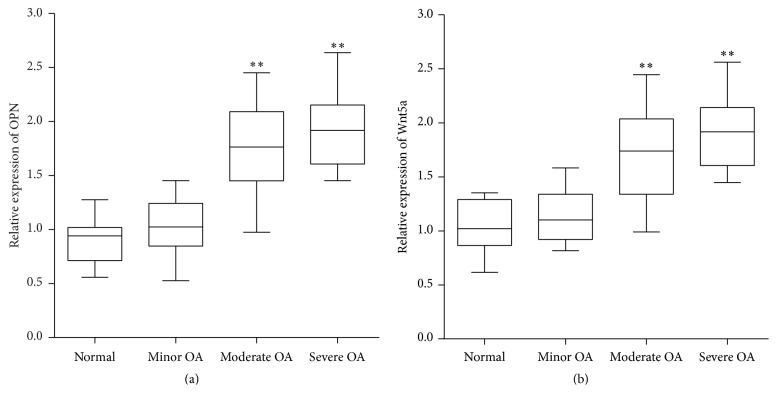
Validation of OPN and Wnt5a expression in OA by real-time PCR. The relative expression was normalized to *β*-actin and ratio to normal group (*∗∗* means *P* < 0.01, versus normal group).

The real-time PCR results showed that the OPN and mRNA levels in different degrees of damaged cartilage tissues were higher than normal tissues and positively related to the severity of OA (Figure 2), which was consistent with immunohistochemistry results.

### 3.3. Each Group's Mankin Grading System

**Figure 3:**
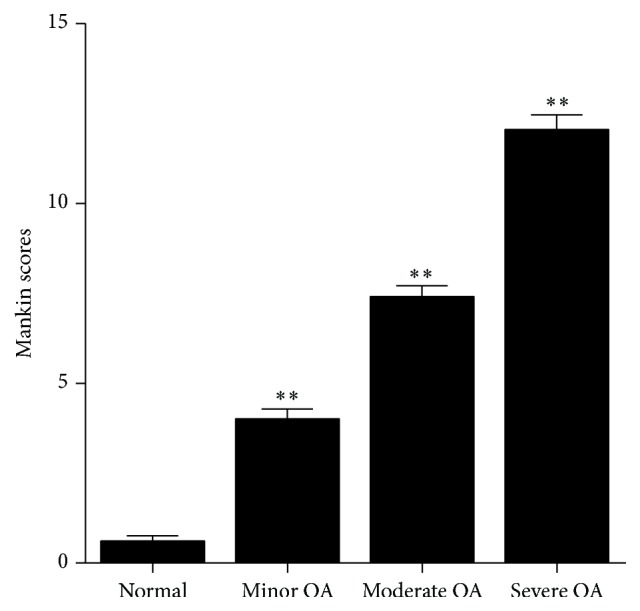
Mankin scores of cartilage in OA and normal group (*∗∗* means *P* < 0.01, versus normal group).

Based on the grading system of Mankin, 60 biopsies in total gained from 10 normal people and 40 patients were ascribed severally to the severe, moderate, minor, and normal groups.  Figure 3 illustrated the Mankin scores of each group.

### 3.4. Improved Expression of OPN Protein in Cartilage Tissues of OA

**Figure 4:**
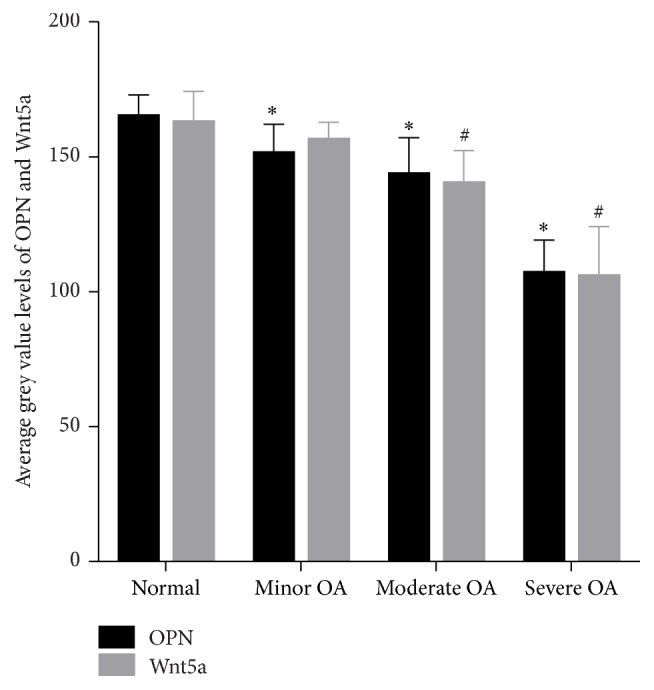
Average grey value levels of OPN and Wnt5a in OA and control group. *∗* means *P* < 0.05, OPN value versus normal group; # means *P* < 0.05, Wnt5a value versus normal group; OPN: osteopontin.

The expression levels of OPN in the different degrees of damaged cartilage tissues were used for comparison (Table 1). OPN, located in the extracellular matrix, was demonstrated to be expressed in the OA and normal groups. In comparison to normal, minor, and moderate groups, a higher expression degree of OPN was shown in the severe group (Figure 4). Higher expression levels are shown in a low grey value. The intercomparison between these groups showed statistical differences (*P* < 0.05). Therefore, according to the results, the OPN protein expression was greatly enhanced in the damaged cartilage.

### 3.5. Enhanced Wnt5a Protein Expression in Cartilage Tissues of OA

The expression degrees in the different levels of impaired cartilage tissues were used for comparison in order to study the Wnt5a function in OA (Table 1). Wnt5a was proved to be shown in the OA and normal groups. Like OPN, the severe group showed higher Wnt5a expression levels in comparison to the normal, minor, and moderate groups (average grey value, 106.50 ± 17.65 versus 140.92 ± 11.43, 157.03 ± 5.76, and 163.58 ± 10.68, resp.; Figure 4). Except comparison between the normal and minor groups (*P* > 0.05), the intercomparisons between the groups were shown to be statistically important (*P* < 0.05).

### 3.6. Correlations between Mankin Scores and OPN and Wnt5a

**Figure 5:**
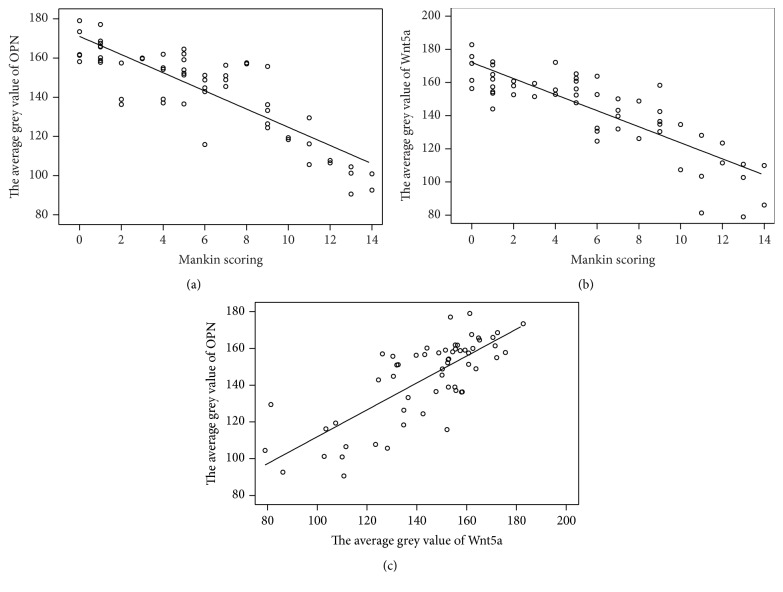
(a) Correlation between the average grey value of OPN and Mankin scoring. Pearson's correlation coefficient, *r* = −0.847 (*P* < 0.01). (b) Correlation between the average grey value of Wnt5a and Mankin scoring. Pearson's correlation coefficient, *r* = −0.843 (*P* < 0.01). (c) Correlation between the average grey values of OPN and Wnt5a. Pearson's correlation coefficient, *r* = 0.769 (*P* < 0.01). OPN: osteopontin.

The software of SPSS was used to evaluate the average grey value for the Mankin scores and every protein in order to study the correlation between OPN and Wnt5a in OA. As for the correlation between the Mankin scores and the average grey value of OPN and Wnt5a, the correlation coefficient of Pearson was considered as *r* = −0.847 (*P* < 0.01) and *r* = −0.843 (*P* < 0.01), respectively. In addition, the correlation between the average grey values of OPN and Wnt5a was considered to have a Pearson's correlation coefficient of *r* = 0.769 (*P* < 0.01) (Figure 5).

## 4. Discussion

Regarding a proinflammatory cytokine, OPN has altered expression involved in pathological conditions, for example, inflammatory disease, cancer metastasis, autoimmune diseases, joint diseases, osteoporosis, and some other stress forms [22]. According to the current research, OPN is important to bone matrix absorption and mineralization progression [23]. Prior studies had demonstrated that the levels of OPN in plasma, articular cartilage, and synovial fluid of OA patients were significantly higher compared to the healthy individuals [6, 7]. In accordance with their results, we observed higher OPN expression levels in severe OA group in comparison to the normal, minor, and moderate groups. In addition, the expression of OPN was proved to relate to level of severity in the degeneration of cartilage. Therefore, we infer that OPN may act as a destructive factor in OA progression and pathology.

Conventionally, Wnt signaling pathways were divided in the *β*-catenin-dependent canonical Wnt signaling pathway and various *β*-catenin-independent noncanonical pathways. Wnt signaling linked to OA incidence via the *β*-catenin-dependent canonical signaling pathway has been approved by majority of studies [16, 24–26]. Zhu et al.'s findings indicated that the *β*-catenin signaling activation in specific chondrocytes of adult mice results in the development of an OA-like phenotype and differentiation of premature chondrocyte which provided definitive and direct evidence about the role that *β*-catenin plays in OA development [16]. In degenerative cartilage, Corr observed increased levels of *β*-catenin, which showed a decreased ability to restrict the function of Wntsignaling to cartilage loss [24].

Wnt5a was considered to be a noncanonical Wnt protein that solely was signaled via noncanonical Wnt signaling. Wnt5a was involved in the early stage of cartilage formation by activating proliferation and inhibiting differentiation of the chondrocyte [27–29]. Church et al. detected that Wnt5a transcripts become restricted to the developing perichondrium of the joint in vivo in the developing chick limb, which contributes to appositional growth [27]. According to Yang et al.'s study, Wnt5a can be gained to regulate the differentiation and proliferation of chondrocyte in Zone I and Zone II, and the change from Zone I to Zone II is inhibited directly by Wnt5a signaling [29]. The mechanism of Wnt5a in OA is complicated and several studies showed that a main catabolic cytokine, interleukin (IL) 1*β*, may be important to destruction of cartilage [30]. The chondrocyte treatment with IL-1*β* upregulated Wnt5a was observed by Ryu and Chun. Conditioned medium from Wnt5a-expressing cells inhibited type II collagen expression, while the reduction of Wnt5a by siRNA inhibited the prohibitive impacts of IL-1*β* on the expression of type II collagen [31]. Ge et al.'s findings also indicated that IL-1*β* upregulates Wnt5a, and the activation of which induced the expression of MMPs 1, 3, 9, and 13 through the signaling pathway of JNK in rabbit temporomandibular joint condylar chondrocytes. The Wnt5a-induced upregulation of MMPs was impaired by the blockage of JNK signaling. Therefore, Wnt5a might be related to the destruction of cartilage by improving the MMPs expression [19].

Few studies reported the relationship between OPN and Wnt5a. In Peng et al.'s study on process of differentiation of human dental papilla cells (HDPCs), OPN was used as a mineralization-related biomarker as its strong mineral binding properties [32]. They found that Wnt5a promoted differentiation of HDPCs by upregulating OPN gene expression. While relationship of them in OA remains unclear, both of their involvement in processes of OA via IL-*β*1 induced signaling pathway in chondrocytes was proved by prior observations, respectively [19, 33]. Our study found that they were highly expressed in osteoarthritic cartilage and correlated to disease severity with the same tendency. Therefore, we infer that OPN may activate NF-*κ*B signaling pathway and finally induce Wnt5a expression via IL-1*β* integrin but still deserve further investigation.

Last but not least, this research's limitations should be acknowledged. First of all, a bigger scale of multiple-center study and investigation are required before a definitive summary is made. In the second place, we only investigated knee OA patients from Xiangya Hospital, Central South University. In the third place, this research was cross-sectional in design and, thus, no summaries about effective relationships and cause can be made.

In conclusion, the correlation between Wnt5a and OPN expression in articular cartilage might be important to the OA progression and pathogenesis. Nonetheless, to elucidate the contribution of Wnt5a and OPN in the pathogenesis of the OA degenerative process, more researches are needed.

## Figures and Tables

**Table 1:** Average grey value levels of OPN and Wnt5a in OA and control group (mean ± SD).

Group	Sample (*n*)	OPN value	Wnt5a value
Normal	13	165.78 ± 7.12	163.58 ± 10.68
Minor OA	18	152.11 ± 9.92^a^	157.03 ± 5.76^d^
Moderate OA	17	144.24 ± 12.78^ab^	140.92 ± 11.43^ab^
Severe OA	12	107.76 ± 11.36^abc^	106.50 ± 17.65^abc^

^a^ *P* < 0.05, versus normal group; ^b^ *P* < 0.05, versus minor group; ^c^ *P* < 0.05, versus moderate group; ^d^ *P* > 0.05, versus normal group; OPN: osteopontin.
